# Myosite focale de la cuisse

**DOI:** 10.11604/pamj.2017.26.200.11740

**Published:** 2017-04-13

**Authors:** Zeineb Alaya, Walid Osman

**Affiliations:** 1 Service de Rhumatologie, Hôpital Farhat Hached, Sousse, Tunisie; 2 Service d’Orthopédie, Hôpital Sahloul, Sousse, Tunisie

**Keywords:** Myosite focale, cuisse, IRM, biopsie musculaire, Focal myositis, thigh, MRI, muscle biopsy

## Image en médecine

Madame M.S âgée de 55 ans, a consulté pour tuméfaction douloureuse de la cuisse droite évoluant depuis six mois faisant suite à une chute de sa propre hauteur. L’examen clinique a montré une cuisse droite tendue sans signes inflammatoires locaux avec une circonférence augmentée de 5 cm par rapport au coté gauche. Le bilan inflammatoire et les enzymes musculaires étaient corrects et les AAN étaient négatifs. La radiographie du fémur droit n’a pas montré d’anomalies en dehors de l’épaississement des parties molles. L’écho-doppler des membres inférieurs était normal. L’IRM de la cuisse droite a montré une discrète atrophie musculaire portant sur le muscle quadriceps essentiellement au niveau du 1/3 moyen et inférieur en T1 (A) avec une plage d’hyper signal STIR hétérogène des vastes médial et latéral qui prend le contraste (B). L’histologie de la biopsie musculaire a confirmé le diagnostic de myosite focale. La patiente a été mise sous antalgiques avec une corticothérapie à faible dose avec amélioration des myalgies et de la différence de circonférence des 2 cuisses de 2 cm à 3 mois de suivi.

**Figure 1:**
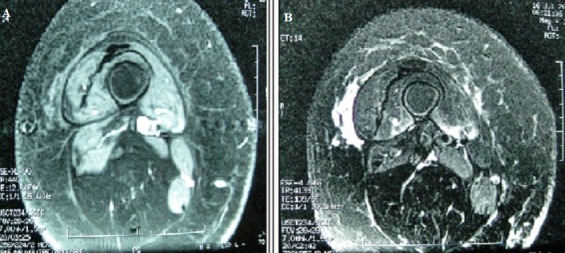
L’IRM de la cuisse droite a montré une discrète atrophie musculaire portant sur le muscle quadriceps essentiellement au niveau du 1/3 moyen et inférieur en T1 (A); avec une plage d’hyper signal STIR hétérogène des vastes médial et latéral qui prend le contraste (B)

